# The importance of assessing vision in falls management: A narrative review

**DOI:** 10.1097/OPX.0000000000002222

**Published:** 2025-02-03

**Authors:** Jignasa Mehta, Aishah Baig

**Affiliations:** 1 School of Allied Health Professions and Nursing, University of Liverpool, Liverpool, United Kingdom; 2 School of Medicine, University of Nottingham, Nottingham, United Kingdom; ∗ jigs@liverpool.ac.uk

## Abstract

**SIGNIFICANCE:**

A comprehensive falls assessment should include the assessment of key visual risk factors, namely, visual acuity, contrast sensitivity, and stereoacuity, to help prevent further falls in older adults. As a minimum, a thorough visual history and uniocular visual acuity assessment would allow appropriate onward referral for intervention.

**PURPOSE:**

Falls prevention is a global public health challenge. The etiology of falls is often complex due to multiple interacting risk factors contributing to postural instability in older adults. Despite national recommendations, the assessment of visual function is often overlooked in falls management. This may be due to a lack of clear guidance on key visual functions that need assessing in this patient group, professional roles, and responsibilities. This review gives an overview of visual risk factors for falls in older adults without cognitive impairment. It focuses on visual functions that can be assessed practically using standard clinical procedures. Possible test selection for a falls clinic or inpatient setting is discussed to help inform the implementation of vision assessments in falls management.

**METHODS:**

Literature searches were conducted on Web of Science (1898 to current), MEDLINE (1946 to present), and APA PsycInfo (1887 to present) using relevant search terms and Boolean operators related to visual functions and falls. Retrospective and prospective studies including randomized controlled trials, observational, cohort, case-control, and qualitative studies were included.

**RESULTS:**

Visual functions decline with age due to the normal aging process and age-related pathologies. Despite considerable heterogeneity across studies, the evidence supports the association of falls with declines in visual functions, including visual acuity, binocular single vision, and the visual field, but most notably contrast sensitivity and stereopsis. Existing vision screening tools, which assess multiple visual functions, are reviewed in light of their usefulness in falls.

**CONCLUSIONS:**

We recommend a vision assessment in the management of falls, which considers visual functions associated with falls, particularly contrast sensitivity and stereopsis. Existing vision screening tools could be adapted or developed for use in falls clinics or the inpatient setting. Eye health professionals should form part of falls multidisciplinary teams or offer training in assessing vision and help to develop intervention pathways for timely management of visual impairment.

Falls prevention is a global public health challenge. The etiology of falls is often complex, due to multiple interacting risk factors, which contribute to postural instability in older adults. Postural stability is achieved through visual, vestibular, and somatosensory input; processed by the cortex; and affected by coordinated motor responses from the muscles, joints, and reflexes.^1^ Inadequate sensory information can therefore affect stability and potentially impact the risk of falling.^2^ Performance of the visual system can be assessed through the measurement of various visual functions, including visual acuity (VA), contrast sensitivity, the visual field, and binocular single vision, that is, the ability to use both eyes simultaneously so that each eye contributes to a common single perception. Visual function has been reported to decline during later life,^3,4^ and although this decline may be due to age-related eye diseases, it could also be due to the normal aging process.^4^ In an effort to reduce the risk of falls, international guidance recommends a multifactorial assessment, including an assessment of vision, in falls services.^5^ Despite this recommendation, the assessment of vision is often overlooked, which may be due to the lack of clear guidance on which visual functions to test and how to test these.

This narrative review adds recently published data on the impact of visual function on falls to the body of evidence^6,7^ and importantly focuses on visual functions that can be practically assessed using standard clinical procedures and existing vision screening tools. The findings will help inform and guide vision assessments and management of visual risk factors for falls.

## METHODS

Literature searches were conducted on Web of Science (1898 to current), MEDLINE (1946 to present), and APA PsycInfo (1887 to present) using relevant search terms and Boolean operators related to visual functions and falls. Studies published in English and with populations 60 years or older were included in the review. Retrospective and prospective studies including randomized controlled trials (RCTs), observational, cohort, case-control, and qualitative studies were included in the review. Studies that included cognitively impaired participants or participants with Parkinson's/or other neurological deficits, however, were not included in the review due to the confounding nature of these conditions on falls, and the aim was to review the literature on vision as a falls risk factor. Reference lists from individual journal articles were manually searched for further relevant sources. Data extracted from each study included outcome measures defined as hip fractures, single and multiple falls, and associated visual function risk factors, that is, VA, contrast sensitivity, depth perception, and visual fields. Adjusted odds ratios are reported where available as a measure of an independent predictor of falls risk. Visual acuity, contrast sensitivity, binocular single vision, depth perception, and visual field are discussed within this review, with a summary of visual screening tools available to assess these visual functions. The practicality of vision tests for use in a falls clinic and inpatient environment was considered, particularly in relation to portability, efficiency, and validity of tests.

### Visual acuity

Studies have reported an increase in prevalence of visual impairment with age, defined as either >6/12 or >6/18 in the better eye.^8–11^ With an increased risk of falls with age, reduced vision becomes a potential risk factor. A review of the risks and types of injuries associated with visual impairment determined that individuals with reduced VA were 1.7 times more likely to suffer a fall, 1.9 times more likely to suffer multiple falls, and 1.3 to 1.9 times more likely to suffer a hip fracture.^12^ There is considerable heterogeneity in the methodology and results of the studies investigating the association between VA and falls. This is due to differences in VA measurements, thresholds set for defining impaired vision, and outcomes, for example, hip fracture^13–15^ or multiple falls.^16–18^

An increased risk of hip fracture has been significantly associated with reduced binocular VA of >6/12,^19^ ≥6/15,^14^ and >6/18.^13^ Although the EPIDOS study was a large cohort study (N = 7575),^14^ the results related to women 75 years and older, compared with adults 60 years and older included in the study reported by Ivers et al.^13^ (cases, N = 911; controls, N = 910), and Snellen VA was tested at 5 m. Table 1 outlines key published studies investigating the association between VA and falls or hip fractures. Although many of the epidemiological studies boast large samples, visual function was normally measured at baseline when prospectively looking at the association of falls with visual risk factors and not reported to be monitored throughout the follow-up period.^14,22^ Potentially, this would not account for refractive or ophthalmic status changes, for example, ocular comorbidities, at the time of the fall. Also, in other studies, participants were asked to recall the number of falls or fractures in the year prior to the baseline vision assessment^15,17,23,25,26,28^; thus, data may be confounded by recall bias and lack of visual status data at the time of the fall or fracture.

**TABLE 1:** Key published studies investigating the association between visual acuity and falls or hip fractures

Authors (year of publication), study design	Sample size and age	Main findings (p values and test used stated where available)
Tinetti et al. (1988),^20^ prospective cohort	Adults ≥75 y, n = 336	RR, 1.4 (95% CI, 0.9–2.0) ≥20% VA loss and 1+ fall Snellen chart
Nevitt et al. (1989),^16^ prospective cohort	Adults ≥60 y, n = 325	RR, 1.5 (95% CI, 1.1–2.1) VA ≥6/15 and 2+ falls Bailey-Lovie chart
Campbell et al. (1989),^21^ prospective cohort	Adults ≥70 y, n = 761	Women: RR, 1.3 (95% CI, 0.8–2.2) Men: RR, 1.3 (95% CI, 0.8–2.8) VA ≥6/12 and 1+ fall Snellen chart
Felson et al. (1989),^22^ the Framingham Study, retrospective cohort	Adults 64–80 y, n = 2633	RR, 1.73 (95% CI, 1.13–2.65) (adjusted); ≥6/9.5 either eye and hip fracture RR, 2.17 (95% CI, 1.24–3.80; p<0.1) (adjusted); ≥6/30 in at least one eye RR, 1.94 (95% CI, 1.13–3.32) (adjusted), ≤6/7.5 one eye and 6/9.5–6/24 in the other eye Snellen chart
Dargent-Molina et al. (1996),^14^ EPIDOS study prospective cohort	Women ≥75 y, n = 7575	RR, 1.9 (95% CI, 1.1–3.1) VA ≥6/15 and hip fracture Snellen chart
Ivers et al. (1998),^23^ Blue Mountains study, cross-sectional	Adults ≥49 y, n = 3299	PR, 1.9 (95% CI, 1.2–3.0) (fully adjusted); p=0.051 for trend VA >6/9 and 2+ falls logMAR chart
Ivers et al. (2000),^13^ Auckland Hip Fracture Study, case-control	Adults ≥60 y Cases: 911 Controls: 910	OR, 1.5 (95% CI, 1.1–2.0) (multiply adjusted); p=0.007 for trend VA >6/19 and hip fracture Snellen chart
Lord and Dayhew (2001),^24^ prospective cohort	Adults 63–90 y, n = 156	RR, 1.59 (95% CI, 0.85–2.98) (adjusted) VA ≥6/10 and 2+ falls logMAR chart
Ivers et al. (2003),^15^ Blue Mountains study, prospective cohort	Adults ≥49 y, n = 3654	HR, 8.4 (95% CI, 1.5–48.5); p=0.017 VA >6/19 and hip fracture logMAR chart
Klein et al. (2003),^17^ Beaver Dam Study, cross-sectional	Adults 43–84 y, n = 3722	OR, 2.02 (95% CI, 1.13–3.63); p=0.01 for trend VA ≥6/12 and 2+ falls ETDRS chart
Coleman et al. (2004),^18^ prospective cohort	Women ≥65 y, n = 2002	OR, 1.43 (95% CI, 1.17–1.75); p=0.0004) Loss of ≥10 letters and 2+ falls Bailey-Lovie chart
Lamoureux et al. (2008),^25^ The Singapore Malay Eye Study, Cross-sectional	Adults 40–80 y, n = 3280	OR, 2.1 (95% CI, 1.4–3.1) VA ≥1.0 logMAR in one eye and >0.3 to <1.0 in the other eye and a fall logMAR chart
Yip et al. (2014),^26^ EPIC-Norfolk Eye study, cross-sectional	Adults 48–92 y, n = 8317	OR, 1.52 (95% CI, 1.17–1.97); VA >6/12 and 1+ falls OR, 1.52 (95% CI, 1.26–1.84); self-reported vision and 1+ falls logMAR chart
Loriaut et al. (2014),^19^ case-control study	96 cases, 60–99 y 103 controls, 62–98 y	OR, 6.4 (95% CI, 3.8–10.8) VA >6/12 and hip fracture Snellen chart
Mehta et al. (2022),^27^ case-control study	Adults ≥60 y 83 cases 83, controls	p=0.018 VA ≥ +0.30 logMAR and a fall ETDRS chart
Gupta et al. (2022),^28^ Singapore Epidemiology of Eye Disease Study, prospective cohort	Adults ≥60 y, n = 1972	OR, 4.32 (95% CI, 2.01–9.28); p<0.001) VA >0.3 in one eye and >0.48 in the other eye and 2+ falls logMAR chart

CI = confidence interval; EDTRS = Early Treatment of Diabetic Retinopathy Study; HR = hazard ratio; logMAR = logarithm of the minimum angle of resolution; OR = odds ratio; PR = prevalence ratio; RR = relative risk; VA = visual acuity.

An increased risk of multiple falls, but not single falls, has been significantly associated with reduced binocular VA >6/9,^23^ ≥6/10,^24^ ≥6/12,^17^ and ≥6/15.^16^ One study, however, reported that bilateral visual impairment, characterized by VA >6/12, significantly increased the risk of single incident falls and recurrent falls.^28^ This study also found that unilateral visual impairment significantly increased the risk of recurrent incident falls.^28^ Impaired VA with an interocular disparity may be a risk factor for falls as this has an impact on depth perception.^29^

The Early Treatment of Diabetic Retinopathy Study may be suitable to test VA in a falls clinic setting. However, if impractical, due to size and limited portability in an inpatient setting, the crowded Keeler logMAR flipbook can be used instead. This test has not been used in falls studies, but the two tests have been found to produce equivalent results in adults.^30^ Instead of a formal test of VA, self-reported vision has also been found to be associated with falls risk. An epidemiological study of 8317 participants investigated the association between measured VA and self-reported vision, in relation to falls.^26^ The authors reported greater odds of falling in individuals with VA >6/12 in the better eye and similarly in those with poor self-reported vision. The association between self-reported vision and falls remained significant after adjusting for VA. This suggests that participants consider other aspects of visual function when reporting on their vision. This may include contrast sensitivity, depth perception, or the visual field.

Overall, there is evidence suggesting an association between binocular VA >6/9 for multiple falls^15^ and between 6/12 and 6/19 for risk of hip fracture.^13–15,19^ The likelihood of falling may be further increased when visual impairment is disparate between the two eyes.^28,29^

### Contrast sensitivity

Visual acuity measures an individual's ability to discriminate high spatial frequency details at high contrast. However, when negotiating a real-world environment, individuals need to perceive objects of varying spatial frequencies, in various contrast conditions, to prevent falls and trips.

Contrast sensitivity has been reported to decline with age^3,31,32^ and age-related eye conditions^33–36^ (Rosen R, et al. IOVS 2015;56:ARVO E-Abstract 2224) associated with falls and hip fractures, such as cataracts, uncorrected refractive error, and age-related macular degeneration (AMD).^19,22,37–43^ The level of contrast sensitivity impairment has been found to be similar to or even greater than that of VA in these conditions.^33,44,45^ Contrast sensitivity also appears to correlate with subjective visual impairment and vision-related quality of life better than VA, particularly in those with cataracts and AMD.^46–50^ Therefore, contrast sensitivity may be a risk factor for falls alone and important to measure in addition to VA. Reduced VA has not been found to be associated with impaired gait,^51^ whereas reduced contrast sensitivity at lower spatial frequencies (1.5 and 3 cpd) has been significantly associated with shorter stride length (p=0.001),^51^ which has been linked to increased risk of falls.^52^

Similar to studies that have investigated the relationship between VA and falls, there are considerable differences in testing methodology and outcomes, making it difficult to compare findings (Table 2). Nonetheless, reduced contrast sensitivity has been found to be a significant risk factor for falls.^24,53^ Fallers were found to perform significantly worse on contrast sensitivity compared with VA testing.^53^ Those with poor low-contrast acuity are also at higher risk of multiple falls than those with poor high-contrast acuity.^24^ A large cohort study (N = 9516) examining risk factors for hip fracture in White women found poor contrast sensitivity (Table 2) and poor depth perception (Table 3) to be significant risk factors over VA, after multivariate adjustment.^54^

**TABLE 2:** Key published studies investigating the association between contrast sensitivity and falls or hip fractures

Authors (year of publication), study design	Sample size and age	Main findings (p values and test used stated where available)
Nevitt et al. (1989),^16^ prospective cohort	Adults ≥60 y, n = 325	RR, 1.5 (95% CI, 1.1–2.1) ≥6/15 and 2+ falls Bailey-Lovie chart
Lord et al. (1991),^53^ cross-sectional	Adults 59–97 y, n = 95	15.1 D (fallers) vs. 17.5 D (nonfallers), p<0.05 1+ falls Melbourne Edge Test
Dargent-Molina et al. (1996),^14^ EPIDOS study, prospective cohort	Women ≥75 y, n = 7575	No significant association between contrast sensitivity and falls. No values presented Vistech VCTS-6500
Cummings et al. (1995),^54^ prospective cohort	Women ≥65 y, n = 9516	RR, 1.2 (95% CI, 1.0–1.5) 1-SD decrease in low-frequency contrast sensitivity and hip fracture Ginsburg Functional Acuity Contrast Test
Ivers et al. (1998),^23^ Blue Mountains study, cross-sectional	Adults ≥49 y, n = 3299	PR, 1.1 (95% CI, 1.0–1.2) (fully adjusted); 3 cpd and 2+ falls PR, 1.2 (95% CI, 1.1–1.3) (fully adjusted); 6 cpd and 2+ falls PR, 1.1 (95% CI, 1.0–1.2) (fully adjusted); 12 cpd and 2+ falls VectorVision CSV-1000
Lord and Dayhew (2001),^24^ prospective cohort	Adults 63–90 y, n = 156	RR, 1.93 (95% CI, 1.01–3.68) ≤18 D at distance and 2+ falls Melbourne Edge Test
Ivers et al. (2003),^15^ Blue Mountains study, cross-sectional	Adults ≥49 y, n = 3654	Contrast sensitivity was not significant in the adjusted model at 2-y follow-up VectorVision CSV-1000
Klein et al. (2003),^17^ Beaver Dam Study, cross-sectional	Adults 43–84 y, n = 3722	OR, 1.63 (95% CI, 1.11–2.39); p=0.02 for trend Contrast sensitivity at 1.5 log units and 2+ falls Pelli-Robson
de Boer et al. (2004),^55^ Longitudinal Aging Study Amsterdam (LASA), prospective cohort	Adults ≥65 y, n = 1509	HR, 1.53 (95% CI, 1.03–2.29); p=0.037 (adjusted); integrated contrast sensitivity score for 1.5, 3, 6, 12, and 18 cpd and 1+ falls HR, 1.66 (95% CI, 1.11–2.48); p=0.013 (adjusted); low spatial frequency contrast sensitivity (1.5 and 3 cpd) and 1+ falls Vistech VCTS_6000-1
Freeman et al. (2007),^56^ prospective cohort	Adults 65–84 y, n = 2375	OR, 0.96 (95% CI, 0.86–1.07) (age-adjusted) (further adjusted data not shown) Contrast sensitivity per 0.3-log unit correct and 1+ falls Pelli-Robson
Mehta et al. (2022),^27^ case-control study	Adults ≥60 y; 83 cases, 83 controls	OR, 1.40 (95% CI, 1.12–1.80); p=0.003 Contrast sensitivity at 18-cpd decrease of 0.15 log units CSV 1000E (and Pelli-Robson)

CI = confidence interval; cpd = cycles per degree; HR = hazard ratio; PR = prevalence ratio; OR = odds ratio; RR = relative risk.

**TABLE 3:** Key published studies investigating the association between stereoacuity and falls or hip fractures

Authors (year of publication), study design	Sample size and age	Main findings (p values and test used stated where available)
Nevitt et al. (1989),^16^ prospective cohort	Adults ≥60 y, n = 325	RR, 1.56 (95% CI, 1.1–2.6) ≥200 inches of arc and 2+ fall Randot stereotest
Cummings et al. (1995),^54^ prospective cohort	Adults ≥65 y, n = 9516	RR, 1.4 (95% CI, 1.0–1.9) Lowest quartile for distant depth perception and hip fracture Howard-Dolman test
Ivers et al. (2000),^13^ Auckland Hip Fracture Study, case-control study	Adults ≥60 y; 911 cases, 910 controls	OR, 6.0 (95% CI, 3.2–11.1); no stereoacuity and hip fracture OR, 3.9 (95% CI, 2.3–6.7); ≥400 inches of arc and hip fracture OR, 4.1 (95% CI, 2.4–7.2); 140 to <400 inches of arc and hip fracture OR, 3.0 (95% CI, 1.7–5.4); >50 to <140 inches of arc and hip fracture Multiply adjusted, p=0.0001 for trend Randot stereotest
Lord and Dayhew (2001),^24^ prospective cohort	Adults 63–90 y, n = 156	RR, 2.26 (95% CI, 1.24–4.14) (adjusted); ≥2.4-cm Howard-Dolman and 2+ falls RR, 1.99 (95% CI, 1.11–3.59) (adjusted); ≥215 inches of arc and 2+ falls Frisby stereotest
Friedman et al. (2002),^57^ The Salisbury Eye Evaluation Study, cross-sectional	Adults 65–84 y, n = 2212	Stereoacuity not a significant predictor of falls. No values presented Randot stereotest
Mehta et al. (2022),^27^ case-control study	Adults ≥60 y; 83 cases, 83 controls	OR, 3.4 (95% CI, 1.20–9.69); p=0.02 110–600 inches of arc and a fall Frisby stereotest

CI = confidence interval; OR, odds ratio; PR = prevalence ratio.

Using the Vistech (Vistech Consultants, Dayton, OH), older people with reduced contrast sensitivity at the mid-lower spatial frequencies of 1.5 and 3 cpd,^55^ or 3.6 and 12 cpd,^23^ were reported to have increased risk of recurrent falls. In contrast, Mehta and colleagues^27^ more recently found that, of all spatial frequencies, impaired contrast sensitivity at 18 cpd was a significant risk factor for falling. The urban world we live in, both indoors and outdoors, consisting of buildings and limited vegetation, is predominantly made up of medium to lower spatial frequency information,^58,59^ whereas the natural world, largely consisting of vegetation and greenery, is made up of mostly higher spatial frequencies.^59^ This may explain the association between impaired contrast sensitivity in the mid-lower spatial frequencies and falls, as often reported mechanisms of a fall include tripping over a rug, curb, or step, usually of a similar color to the lower surface.

The following quote given by a participant of a mixed-methods cohort study captures the perception of the importance of poorly contrasting surfaces in the mechanism of falls: “I was stepping on an escalator, which did not have a yellow line to mark the end of step in the shopping center…. I can't see well on metal escalators and sometimes I don't see things that are right in front of me especially if they are all the same color.”^60^ Qualitative findings were supported by quantitative findings using the Pelli-Robson chart. A 1-unit increase in log contrast sensitivity (20 letters) approximately halved the risk of a fall.^60^

Studies have shown that preprinted test charts such as the FACT test (Ginsburg, AP, PhD, Wright-Patternson Air Force Base, OH), the Vistech, and similar tests have demonstrated ceiling/floor effects and poor test-retest reliability,^61–69^ making it difficult to derive conclusive evidence from results. This may also render them unsuitable for screening purposes in a falls clinic setting, in addition to the length of time required to undertake the tests.

On the other hand, the Melbourne Edge Test^70,71^ and Pelli-Robson^68,69,72,73^ have demonstrated repeatability and test-retest reliability. The Pelli-Robson is the most routinely used clinical test for measuring contrast sensitivity, and age-specific normative values have been published for this test.^74,75^ Reduced contrast sensitivity with the Pelli-Robson chart has been shown to elevate the risk of two or more falls by a factor of 1.63.^17^ Even though the Pelli-Robson test measures contrast sensitivity at a single low spatial frequency of approximately 0.5 to 1 cpd, the association between reduced contrast sensitivity for low spatial frequencies and falls and efficiency and reliability of the test may make it most suitable for a falls clinic setting at present.

The quick quantitative contrast sensitivity function could be an alternative test to measure contrast sensitivity across multiple spatial frequencies efficiently and reliably in a falls clinic or inpatient setting.^76,77^ This test uses a Bayesian active learning algorithm on an electronic platform,^76,78^ which is promising, given the rise of electronic device usage, such as tablets in clinics and inpatient wards.

There is a body of evidence supporting an association between reduced contrast sensitivity and falls, particularly in the mid-lower spatial frequency range. Contrast sensitivity has also been reported to be associated more significantly with falls than VA,^24,54^ but there is a lack of evidence from large-scale epidemiological studies where contrast sensitivity has been a greater risk factor than VA.^14,15,17^ It is therefore advisable to measure both VA and contrast sensitivity in any falls vision assessment.

### Binocular single vision and depth perception

In this review, binocular single vision is defined as the “ability to use both eyes simultaneously so that each eye contributes to a common single perception” as opposed to binocular vision, which is the “simultaneous perception of two images, one from each eye.”^79^ Contrast sensitivity and stereoacuity have been found to be the visual risk factors most associated with postural sway and instability^80,81^ and limitations in walking and climbing stairs in older adults.^82^ Impaired binocular single vision, eye movement disorders, and a decline in stereopsis have been associated with older age.^83–87^ It can be argued that impaired stereopsis or a loss of binocular single vision may increase postural instability and the risk of a fall, due to the diminished perception of depth and presence of diplopia. The association between impaired depth perception and falls is an understudied phenomenon. Nonetheless, all studies in Table 3 but one^57^ suggest that impaired depth perception is a significant risk factor for falls.^13,16,24,27,54^ Different stereotests have been used across studies to investigate the association between impaired depth perception and falls, including the Randot stereotest,^13,16,57^ Frisby stereotest,^27^ and Howard-Dolman test,^24,54^ which is rarely used in clinical settings.

Studies have reported that reduced stereoacuity on the Randot test was strongly associated with multiple falls, but not a single fall^16^ and hip fractures.^13^ Ivers and colleagues^13^ reported a sixfold risk of having a hip fracture if the individual had no demonstrable stereopsis. On the contrary, in the Salisbury Eye Evaluation Study, stereoacuity measured with the Randot test was not found to be a significant predictor of falls.^57^

Using the Frisby test, Mehta et al.^27^ found that stereoacuity worse than 85 seconds of arc was significantly associated with experiencing a fall. The Frisby test has been found to have better test-retest reliability than the Randot.^88^ An advantage of this test is that it can be used in a slight downgaze position to assess depth perception in the lower field. This is particularly useful in studies examining the association of depth perception and falls, as individuals often negotiate stairs and other obstacles in their lower field. Also, the Frisby test has coarse elements, and its pattern tolerates a fair degree of blurring (personal communication with John Frisby), which may occur when older adults are potentially looking through the incorrect portion of their varifocals. The test design is also practical for use in a falls clinic or inpatient setting, due to its small size and portability.

Some studies have inferred poor depth perception based on an interocular difference in VA rather than measuring stereoacuity^22,25,29^ and have found associations with a single fall,^25^ multiple falls,^28^ and hip fractures,^22^ although the level of difference in VA attributed to these associations varies between studies. The Framingham Study (N = 2633) was conducted over 10 years to determine the risk of hip fractures associated with visual impairment.^22^ This study reported that those who had a difference in acuity between both eyes, for example, moderately impaired vision (20/30 to 20/80) in one eye and good vision (better than 20/25) in the other, had a higher risk of fracture (relative risk, 1.94; 95% confidence interval [CI], 1.13 to 3.32) than those with a similar degree of binocular impairment (relative risk, 1.11; 95% CI, 0.55 to 2.24). Similarly, in a study of 3280 Malay adults aged 40 to 80 years, a severe visual impairment in one eye (equal to or worse than 6/60) and mild or moderate visual impairment (worse than 6/12 but better than 6/60) in the other doubled the risk of falls (odds ratio, 2.1; 95% CI, 1.4 to 3.1).^25^ Falls were recorded retrospectively in this population-based cross-sectional study, therefore limiting the generalizability of these findings.

A key RCT, the PROFET study (Prevention of Falls in the Elderly Trial), evaluated the benefit of having a structured interdisciplinary assessment (N = 184) versus usual care (N = 213) in people who have fallen to prevent further falls.^29^ The authors considered the participants to have poor binocular single vision if they had a disparity of two lines or more in acuity between the two eyes. Based on this criterion, they reported 62% (N = 94) of the participants who attended Accident and Emergency following a fall to have poor stereoscopic vision.

Further evidence to support the association between poor binocular single vision and musculoskeletal injury, fractures, and falls was examined in a large 10-year retrospective review of 2,196,881 Medicare beneficiaries.^89^ A binocular single-vision disorder was present in 5% of patients (including a diagnosis of strabismus, diplopia, amblyopia, or nystagmus), and this group had an increased risk of musculoskeletal injury, fracture, or fall after adjusting for confounding factors (odds ratio, 1.27; 95% CI, 1.25 to 1.29; p<0.001).

Fewer studies have investigated reduced stereoacuity and more so the level of binocular single vision, for example, fusional amplitude, as a risk factor for falls. Despite the use of varied stereotests, outcomes, and methods, the evidence reviewed here suggests that reduced stereoacuity is a key visual risk factor for falls. Therefore, testing stereopsis should form part of a falls vision assessment. However, care must be taken to ensure the patient is wearing the appropriate refractive correction for the distance the test is conducted. This is not always explicit in studies that have examined this visual function in relation to falls.^13,54,57^ If unequal VA with appropriate refractive correction is to be used as a marker for poor binocular single vision or impaired depth perception, further robust evidence is required to determine the relationship between level of unequal VA and stereoacuity.

### Visual fields

Peripheral vision plays a key role in guiding our gaze and movement for safe locomotion and navigation in the real world.^90^ It is particularly important for tasks such as climbing stairs or walking, as we require attention directed ahead, in addition to an awareness of what is around us and by our feet.^90^ Visual field sensitivity and size have been found to decrease with older age in normal observers.^91–95^ Peripheral and central visual field defects are often caused by conditions that are more prevalent with age, such as glaucoma, stroke, and AMD. Visual field defects can be a risk factor for falls, particularly if defects obscure obstacles. Unilateral and bilateral visual field losses were associated with a sixfold risk of recurrent falls over a 3-year period.^96^

Using the Humphrey Field Analyzer, both mild and severe visual field losses, characterized as missing 1 to 9 points and ≥20 points, respectively, have been associated with fall-related fractures.^97^ Hip fractures have also been associated with visual field loss of ≥5 points in older adults.^15^ Table 4 highlights the key published studies investigating the association between visual field defects and falls.

**TABLE 4:** Key published studies investigating the association between visual field deficits and falls or hip fractures

Authors (year of publication), study design	Sample size and age	Main findings (p values and test used stated where available)
Ivers et al. (1998),^23^ Blue Mountains study, cross-sectional	Adults ≥49 y, n = 2003	OR, 1.5 (95% CI, 1.0–2.3) (adjusted) p=0.096 for trend 5 points missed and 2+ falls HFA (76-point, 30° program)
Ivers et al. (2003),^15^ Blue Mountains study, cross-sectional	Adults ≥49 y, n = 3654	HR, 5.5 (95% CI, 1.0–29.8); p=0.047 (adjusted) ≥5 points missed and hip fracture HFA (76-point, 30° program)
Freeman et al. (2007),^56^ The Salisbury Eye Evaluation Study, prospective cohort	Adults 65–84 y, n = 2375	OR, 1.08 (95% CI, 1.03–1.13); 10 points missed on binocular VF and 1+ falls OR, 1.05 (95% CI, 1.01–1.09); 5 points missed in central field and 1+ falls OR, 1.06 (95% CI, 1.03–1.10); 4 points missed in peripheral field and 1+ falls HFA (81-point, full-field)
Coleman et al. (2007),^99^ prospective cohort	Women ≥70 y n = 4071	OR, 1.5 (95% CI, 1.11–2.02); p=0.08 (adjusted) ≥20 points missed and 2+ falls HFA (76-point, 30° program)
Coleman et al. (2009),^97^ prospective cohort	Women ≥65 y, n = 4773	HR, 1.40 (95% CI, 1.11–1.78); p=0.006 (fully adjusted); 1–9 points missed and hip fracture HR, 1.46 (95% CI, 1.13–1.89); p=0.004 (fully adjusted); ≥20 points missed and nonspine/hip fracture HFA (76-point, 30° program)
Patino et al. (2010),^98^ Los Angeles Latino Eye Study, cohort study	Adults ≥40 y, n = 3203	OR, 1.42 (95% CI, 1.06–1.91); p=0.02 Moderate to severe VF impairment Mean deviation ≤6 dB and fall HFA (Monocular 24-2 Swedish Interactive Threshold)
Black et al. (2011)^104^ Prospective observational	Adults with glaucoma aged ≥60 y n = 71	RR, 1.57 (95% CI, 1.06–2.32); p=0.024 (adjusted) Inferior VF loss and 1+ fall RR, 1.82 (95% CI, 1.12–2.98); p=0.016 (adjusted) Inferior VF loss and injurious fall HFA (Monocular 24-2 Swedish Interactive Threshold)

CI = confidence interval; HFA = Humphrey Field Analyzer; HR, hazard ratio; OR = odds ratio; RR = relative risk; VF = visual field.

Moderate to severe visual field impairment, defined as a mean deviation of ≤6 dB, has been significantly associated with experiencing a fall.^98^ Missing 5 or more points^23^ or ≥20 points^99^ has also been associated with recurrent falls, but not significantly (p>0.05).

The location of the visual field deficit may also be important when considering visual field loss as a risk factor for falls. When walking, we direct our gaze ahead to plan and avoid distant or oncoming obstacles. However, we also have an awareness of the ground immediately in front of us in the inferior visual field, to negotiate steps and avoid trip and slip hazards, for example. Visual field deficits in the lower field have been associated with poor mobility.^101^ Healthy young individuals artificially restricted from using their inferior visual field while walking on multisurface terrain^102^ or descending a staircase^103^ pitch their head at a more downward angle and step more cautiously to compensate for the loss of visual field. Meanwhile, obscuring the central vision of healthy individuals climbing stairs does not appear to affect behaviors.^100^

Studies have examined specific field losses, for example, inferior or superior in the risk of falls.^56,99,104^ However, they either were not examined as independent contributors to the risk of a fall^99^ or were insignificant predictors.^56^ An association has been reported between a loss of binocular inferior visual field and a greater risk of falls and injurious falls in older adults with glaucoma.^105^ A longitudinal study examining glaucoma patients with 6-monthly visual field tests found that a history of quicker visual field loss (0.5 dB/y) due to glaucoma was associated with an increased risk of falling, in addition to the severity of the visual field loss.^105^ Rapid rather than gradual visual field loss may inhibit the ability of the individual to adapt to and develop compensatory strategies to this impairment.

Automated perimeters such as the Humphrey Field Analyzer are commonly used for visual field testing in the clinical setting. Automated perimetry often involves large devices and is lengthy to perform, making it impractical in a falls clinic and certainly in an inpatient setting. Binocular confrontational visual field testing with a skilled practitioner may be a suitable alternative. Confrontational testing has been shown to demonstrate high specificity and predictive value.^106,107^ The test is particularly sensitive for moderate to dense visual field defects, including homonymous hemianopias and altitudinal defects,^106,107^ which may be useful for detecting inferior field defects. Confrontational testing is not, however, sensitive for detecting mild defects or glaucomatous changes, which may contribute to falls. Portable electronic visual field tests, for the purpose of case finding and monitoring of glaucoma in the community and at home, are in development and being tested for validity.^108,109^

There are methodological differences in the way studies were conducted regarding visual field deficits and falls, for example, variation in thresholds for visual field defects and types of visual field tests performed. However, there is some evidence to suggest an association between visual field loss and hip fracture risk,^15^ as well as falls risk^98^ and progressive glaucomatous visual field loss, particularly in the inferior field.^105^

### Vision screening tools

Many of the standard clinical vision tests mentioned in this review require sufficient expertise to perform and interpret, presenting a barrier to assessing vision in falls patients. Eye health professionals, such as orthoptists, optometrists, or ophthalmic nurses, rarely form part of the falls multidisciplinary team (MDT), which typically includes geriatricians, nurses, occupational therapists, physiotherapists, and pharmacists.

Some validated multifactorial falls risk assessment tools include a formal assessment of visual function that any member of the falls MDT can be taught to perform. These tools include the Stopping Elderly Accidents, Deaths, and Injuries, developed by the Centers for Disease Control and Prevention; Neuroscience Research Australia's FallScreen The Falls Risk calculator; and Neuroscience Research Australia's QuickScreen Clinical Falls Risk Assessment Tool.

The Stopping Elderly Accidents, Deaths, and Injuries risk assessment tool and QuickScreen were designed for use in primary care settings but could be used in other settings. However, distance VA is the only measure of visual function. A Snellen chart is suggested as a possible choice of VA test. If VA is worse than 20/40, the patient is advised a referral to an eye specialist for further examination, and their medication is reviewed, to minimize pharmacologically induced visual side effects. If the patient uses bifocals when walking outdoors, they are recommended single-vision distance glasses instead.^110,111^

QuickScreen includes an assessment of low-contrast (10%) VA as the single assessment of visual function, given its increased predictive value over high-contrast VA.^24^ If the patient is unable to read all of line 16, they are to be offered a vision information sheet; examined for glaucoma, cataracts, and refractive error; and referred to an eye specialist if necessary.^112^

FallScreen has two versions: a short-form screening version developed for use in acute hospital or long-term care settings and a long-form version for use in rehabilitation settings and dedicated falls clinics. In the short form, contrast sensitivity and depth perception are measured, which are the two most important visual risk factors for falls.^27^ Contrast sensitivity is measured using the Melbourne Edge Test and depth perception using a rod alignment test. The long form additionally incorporates binocular high-contrast (similar to a Snellen chart) and low-contrast (10%) VA. Results from these forms are inputted into a computer software, which generates a falls risk score, individualized recommendations, and interventions for reducing falls risk, such as referral to an eye specialist.^112,113^

The Royal College of Physicians “Look Out! Bedside Vision Check” has not been validated for falls prevention in hospitals but aims to identify gross visual impairment, which may be considered a falls risk on the ward.^114^ Patients are recommended a sight test with a local optician if they fail the assessment. The tool involves a brief visual history and assessment of binocular distance and near VA by asking patients to read a sentence/identify two pictures at 6/12, gross ocular motility, and binocular confrontational fields. Measuring VA binocularly could risk neglecting an intraocular difference in VA, contributing to reduced depth perception and falls risk.^22,25,28^ Depth perception itself is not measured in this tool, as well as contrast sensitivity, which have been found to be the most significant visual risk factors for postural instability and falls.^24,80,81^ Any suitably trained member of the ward team can use the tool. However, the most recent Royal College of Physicians “National Audit of Inpatient Falls Annual Report” found that still only 52% of inpatient falls resulting in femoral fractures had an inpatient vision assessment before they fell.^115^ This highlights the need to investigate other barriers to the implementation of assessing vision in falls management.

In addition to falls-specific vision screening tools, there are generic tools available to assess multiple visual functions, which could be adapted to a falls population. The City Vision Screener^116^ is a customizable computer software that can be used to test various visual functions, including high- and low-contrast VA, stereopsis, and visual field. It is also practical if used with a laptop in a falls clinic or inpatient setting, in comparison to other automated vision screening devices, which may not be as portable or as easily accessible. The combination of testing high- and low-contrast VA, using this screener, has been found to be highly sensitive and specific for detecting correctable vision impairment in older adults.^117^

The Thomas Pocklington “Eyes Right Toolkit” Flipchart Vision Screener (FVS) also detects gross visual impairment but not specifically for the purposes of falls prevention. Therefore, it does not test all visual functions associated with falls. It is designed for use in any setting, particularly in the community, and can again be performed by any suitably trained individual. The FVS assesses binocular near vision (N6 to N14 at a comfortable reading distance) and monocular distance vision at high contrast (0.2 to 0.6 logMAR at 3 m) and low (10%) contrast (0.4 or 0.3 to 0.7 logMAR at 3 m); however, stereopsis and visual fields are not tested. If the patient fails any part of the assessment, he or she is advised a sight test at a local optician. Unlike the “Bedside Vision Check,” the FVS has been evaluated against the City Vision Screener and found to be specific and sensitive for the detection of correctable visual impairment in the community.^117^

The Visual Impairment Screening Assessment is a validated assessment tool that can be used in a printed version or as a software application.^118^ The tool is used to detect visual impairment following brain injury, including stroke. The assessment can be undertaken by any trained member of the rehabilitation team and involves the following: a brief history and uniocular near and distance VA (0.0, 0.2, 0.4, 0.6, and 0.8 logMAR at 3 m and N5, N6, N8, N14, and N18 at 35 cm). Ocular movement testing and convergence, binocular visual fields to confrontation, and visual attention are also assessed. Near VA, convergence, and visual attention may not be necessary to test in the falls population, but contrast sensitivity and stereopsis are, which are not assessed within this tool. Those with detected visual impairment are referred to an orthoptist directly for further assessment and management.

Table 5 highlights the aspects of vision independently associated with falls risk and their most practical means of assessment for a falls clinic or inpatient setting. The majority of this assessment would be possible only in a cognitively normal population. The Visual Impairment Screening Assessment tool can be adapted to assess VA in those with cognitive impairment using preferential looking. The same adaptation could be used for this population. A software application for a tablet may aid usability and practicality for both a falls clinic and ward setting, as it would remove the need for transporting several tests, some of which may be less portable. It is also essential that the appropriate refractive correction is worn for the distances at which each of the visual functions is assessed.

**TABLE 5:** Visual risk factors for falls, practical assessment methods for a falls clinic, or inpatient setting and management options

Visual risk factors	Method(s) of assessment
Uniocular distance visual acuity	• Crowded Keeler logMAR flipchart or • VISA or FVS method and • VA tested with and without pinhole • Ask about cataract-related symptoms
Stereopsis/depth perception	• Frisby stereotest or • Reduced depth perception could be inferred from an interocular visual acuity difference and • Ask about presence of diplopia
Binocular visual field	• Binocular visual fields to confrontation
Uniocular contrast sensitivity	• Electronic application utilizing qCSF method or • Low-contrast VA, FVS, or QuickScreen method

FVS = Flipchart Vision Screener; logMAR = logarithm of the minimum angle of resolution; qCSF = quick contrast sensitivity function; VA = visual acuity; VISA = Vision Impairment Screening Assessment.

Based on associations with falls risk, multiple aspects of vision could be assessed in falls prevention practice to identify vision-related risk factors for falls, although expertise in assessing vision and specialized equipment are required. Although the screening methods and measures have not been evaluated as part of this review, it is an area of future research to determine their independent predictive value in the context of the multifactorial nature of risk factors for falls in older people.

### Management of visual risk factors for falls

The management of patients with impaired visual functions, such as VA, contrast sensitivity, depth perception, or visual field, will be guided by the case history. This will help determine if the visual impairment is due to a recent-onset ophthalmic condition (e.g., cataracts, uncorrected refractive errors) or a long-standing condition (e.g., amblyopia or inherited ophthalmic conditions).

If the practitioner determines that impaired visual functions are not long-standing or asymptomatic, the patients need to be referred for further ophthalmic investigations to determine if they are due to correctable conditions, such as uncorrected refractive error or cataracts. In the absence of any treatable cause, patients should be referred to low-vision services, which may include involvement of occupational therapists and rehabilitation teams in the community. These teams provide education, support, strategies, and equipment for managing visual impairment in daily living.

Uncorrected refractive error and cataracts are two of the most common visual diagnoses associated with fragility hip fractures^22,38,39,42^ and falls.^23,43^ A meta-analysis found that timely first eye cataract surgery significantly reduced the risk of recurrent falls.^37^ Although a reduction (33%) in the falls incidence has been reported following first eye cataract surgery, this needed to be accompanied with a less than 0.75 D change to the spectacle power of the operated eye.^43^ Cumming et al.^119^ also reported that the provision of glasses did not reduce falls in their RCT but acknowledged that the control group data were potentially confounded by the control participants seeing an eye professional in the follow-up period. Nonetheless, any changes to refractive corrections need to be managed carefully, particularly if it is a significant change. Similarly, the use of multifocal lenses has been linked to an increased risk of falls and in particular trips,^120,121^ compared with when wearing distance single-vision spectacles.^122^ However, this needs to be explored with the patient during the consultation, as it has been shown in an RCT that active older adults who take part in frequent outdoor activities and have considerable correctable distance refractive error should use single-lens distance glasses. Although those who have considerable distance refractive error but take part in little outdoor activity should use multifocal lenses.^123^

It is advisable that the chosen visual assessment tool and intervention pathway should distinguish between individuals who need to attend an optician for management of refractive error and those requiring timely referral to an ophthalmology service for a more comprehensive eye examination and initiation of treatment for other ophthalmic conditions, such as cataracts.^42^ To distinguish between the likelihood of refractive error or other pathology, VA would need to be measured with and without pinhole.^42^ Pinhole acuity has been shown to have good sensitivity for detecting refractive error.^124^ A patient history could also incorporate asking about specific visual symptoms related to cataracts. Contrast sensitivity may be reduced to a greater extent than VA in the presence of cataracts and may help to explain symptoms and support intervention.^33,45^ Patient history should also determine the presence of binocular diplopia. Symptomatic patients could again be referred directly to an ophthalmic service, where prisms, occlusion, or orthoptic exercises may be issued to manage the diplopia and therefore potentially reduce the risk of falls.

## CONCLUSIONS

There is considerable heterogeneity across the design, reporting, and results of studies that have investigated the association of falls and visual risk factors. Additionally, due to the abundance of and interaction between falls risk factors, investigating the association between impaired vision and falls is challenging. There is a body of evidence to support the association of falls with deficits in visual functions, including VA, binocular single vision, and the visual field, but most notably contrast sensitivity and depth perception.

The assessment of visual function is often overlooked in falls services. This may be due to the lack of clear guidance on which visual functions to test, how they should be tested, what constitutes an abnormal result, and roles and responsibilities. The findings from this narrative review have outlined the key visual risk factors for falls that need assessing: VA, contrast sensitivity, and stereoacuity. Uniocular assessment of VA as a minimum could potentially highlight issues with stereoacuity. An existing vision screening tool could be adapted for use in falls patients by considering visual functions associated with falls, including contrast sensitivity and stereopsis. Age-related declines in visual function are expected but may be due to treatable conditions that could reduce risk of falls if managed promptly.

We recommend that eye health professionals, such as orthoptists, should either form part of the falls MDT or offer training to the MDT in assessing vision, in the same way that orthoptists are involved in the training of other health professionals to conduct school-aged vision screening and vision screening in stroke patients. Collaboration should extend to developing appropriate referral criteria for detected visual abnormalities, local intervention pathways, and auditing data.
